# Beyond the Expected: A Case‐Based Exploration and Literature Review on the Rare Evolution of Pseudomyxoma Peritonei From a Side‐Branch Intraductal Papillary Mucinous Neoplasm

**DOI:** 10.1155/cris/2954221

**Published:** 2026-06-12

**Authors:** Noah S. Brown, Drew Ferguson, Marshall Baker, Courtney L. Scaife

**Affiliations:** ^1^ Department of Surgery, University of Utah, Salt Lake City, Utah, USA, utah.edu; ^2^ Department of Pathology, University of Utah, Salt Lake City, Utah, USA, utah.edu

**Keywords:** case presentation, IPMN, pseudomyxoma peritonei

## Abstract

**Objectives:**

We present a case of a ruptured side‐branch intraductal papillary mucinous neoplasm (IPMN) who was found to have pseudomyxoma peritonei (PMP) with a deposit of acellular mucin in the pelvis.

**Case Presentation:**

The patient is a 65‐year‐old female who initially presented to care with intermittent right upper quadrant pain and fullness. Preoperative imaging demonstrated a large cystic mass in the pancreatic tail, and, therefore, she was taken to the operating room for a distal pancreatectomy and splenectomy. Intraoperatively, she was found to have two distinct deposits of acellular mucin. Both deposits were nonadherent to the surrounding tissue and removed.

**Results:**

The patient recovered postoperatively without incident. She will be surveilled postoperatively with serial computed tomography.

**Conclusion:**

PMP arising from an IPMN of the pancreas is a rare and potentially devastating complication of the disease. Given the complexity of these cases, a multidisciplinary discussion for ongoing management is essential, and these cases are best managed at centers with significant experience in the management of PMP from other sources.

## 1. Introduction

Pseudomyxoma peritonei (PMP) is a rare and severe manifestation of several abdominal malignancies. Tumors of the appendix, ovaries, and colon or rectum are the most common primary tumor sources. A much less common neoplasm that can cause PMP is the intraductal papillary mucinous neoplasm (IPMN) of the pancreas. The development of PMP either concurrently with or as a recurrence following the treatment of IPMN has been reported only 19 times in the literature with an overwhelmingly male predominance. We present one such case in a female with a review of the literature to highlight this rare phenomenon.

## 2. Case Presentation

Our patient is a 65‐year‐old, otherwise healthy female who was evaluated by her primary care physician for a several‐month history of intermittent right upper quadrant pain and fullness. As part of her evaluation, an abdominal ultrasound was ordered, which incidentally demonstrated an 8.0 cm × 8.4 cm cystic mass in the tail of the pancreas. This was followed by an MRCP demonstrating an 8.2 cm × 7 cm × 7.1 cm cyst involving the tail of the pancreas (Figure 1). No dilation of the main pancreatic duct or communication between the cyst and the pancreatic duct was noted. She was referred to gastroenterology for endoscopic ultrasound and fine‐needle aspiration. Pathology from the FNA demonstrated an abundance of extracellular mucin without any evidence of high‐grade dysplasia. Due to fluid viscosity, an adequate sample size for amylase testing was unable to be obtained, and therefore, the FNA specimen was not evaluated for amylase content. Given the size of the lesion, she was referred to our clinic for further workup. A CT pancreas protocol was obtained, again demonstrating the cystic lesion, now 8 cm × 8.2 cm in size (Figure 2). No dilation of the main pancreatic duct or communication between the cyst and the pancreatic duct was noted. Laboratory evaluation was significant for an elevated carcinoembryonic antigen (CEA) of 4.7 and an elevated cancer antigen 19‐9 (CA 19‐9) of 98.

**Figure 1:**
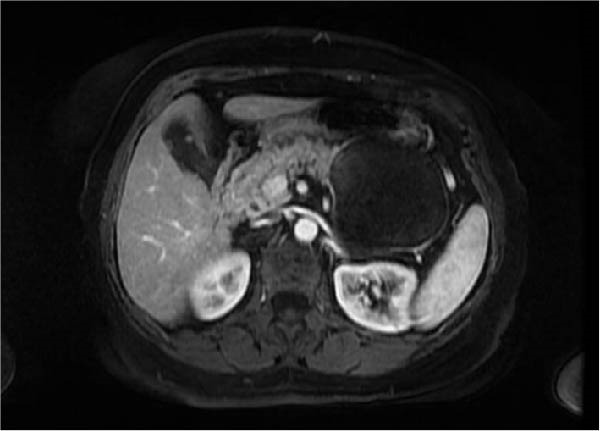
Representative liver acquisition volume acceleration sequence image from preoperative T1‐weighted contrast‐enhanced MRI demonstrating a large thin‐walled cystic lesion abutting the tail of the pancreas. No pancreatic ductal dilation was noted.

**Figure 2:**
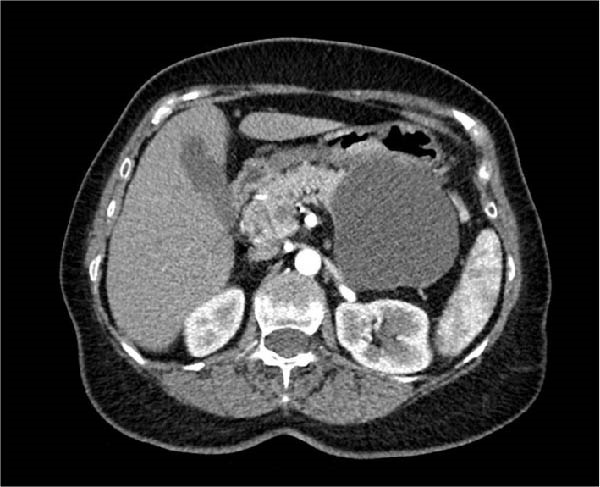
Preoperative arterial phase CT imaging demonstrating a thin‐walled, predominately cystic lesion involving the pancreatic tail.

She was taken to the OR for a laparoscopic‐assisted distal pancreatectomy and splenectomy. Intraoperatively, two deposits of mucin separate from the intact tumor were noted. Neither of these deposits had been visualized on preoperative imaging, and both measured <5 mm. One deposit was in the lesser sac adjacent to the tumor and a second deposit was noted in the pelvis. Both deposits were not adherent to the surrounding tissue, removed with suction, and sent to pathology for evaluation. The remainder of the case proceeded without incident. Pathology evaluation demonstrated a cyst lined with mucinous epithelial cells with predominately low‐grade dysplasia. There were scattered areas of epithelial tufting with loss of nuclear polarity consistent with focal high‐grade dysplasia (Figure 3). Immunohistochemical stains for estrogen receptor were performed on multiple tissue blocks to identify possible ovarian‐type stroma, all of which were negative (Table 1). The lack of ovarian‐type stroma leads away from the diagnosis of mucinous cystic neoplasm. Therefore, the lesion is favored to be a side‐branch IPMN with focal high‐grade dysplasia. Acellular mucin was noted to extend beyond the capsule of the lesion and involved the inked surface defect (Figure 4). The extrapancreatic deposits were both found to be acellular mucin surrounded by reactive mesothelial cells (Table 1). The patient recovered without complication and was discharged home on postoperative day 3. She was seen in the clinic 3 months postoperatively, where she reported a strong recovery with no concerns. We obtained baseline surveillance scans, which demonstrated no recurrent pancreatic lesions or peritoneal fluid. Repeat tumor markers obtained 3 months postoperatively returned to normal levels with a CEA of 1.2 and CA 19‐9 of 33. We intend to surveil the patient with a CT abdomen and pelvis every 6 months moving forward. No written consent has been obtained from the patients, as there is no patient‐identifiable data included in this case report. This case was reported in accordance with the CARE consensus‐based clinical case reporting guidelines and is presented with the CARE Checklist (File S1).

**Figure 3:**
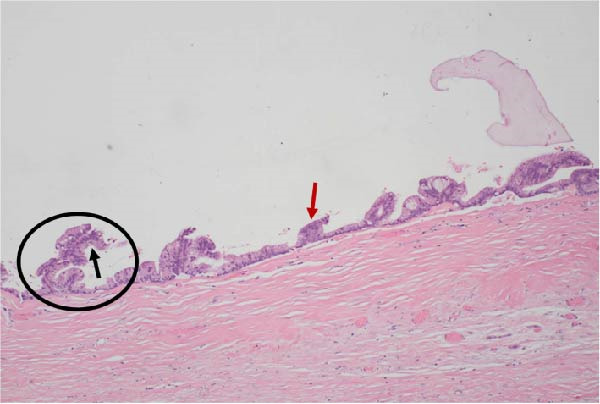
Hematoxylin and Eosin (H&E) stain demonstrating a low‐power view of mucinous epithelium with high‐grade dysplasia indicated by the architectural tufting (black circle), nuclear overlapping (black arrow), and loss of basal nuclear polarity (red arrow). Image was captured with a 10× objective.

**Figure 4:**
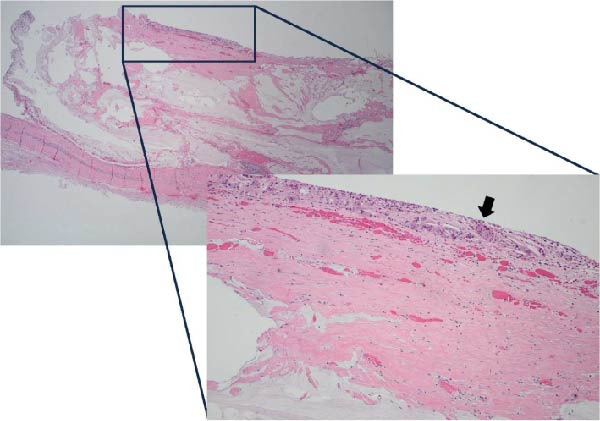
Hematoxylin and Eosin (H&E) stain demonstrating a low‐power view of a fibrous capsule with dissecting blue–gray mucin throughout (left). Along the capsular surface is an area of reactive mesothelial cells and foreign body histiocytic giant cell reaction in response to the ruptured and extravasated mucinous cyst contents (black arrow), image blown up on right. Image was captured with a 2× and 10× objective.

**Table 1:** Summary of immunohistochemical stains performed.

Immunohistochemical stain	Specimen	Result
Estrogen receptor	Distal pancreatectomy and splenectomy	Negative
BerEP4	Pelvic mucin deposit	Negative
Calretinin	Pelvic mucin deposit	Positive

## 3. Discussion

IPMN is a rare premalignant condition comprising ~10%–13% of pancreatic cysts, 25% of cystic neoplasms, and 1% of pancreatic carcinomas [1]. First described in 1982, IPMNs consist of epithelial cells growing from the pancreatic ductal system and producing mucin [1, 2]. A typical “adenoma‐carcinoma” sequence is observed in IPMNs with low‐grade dysplasia progressing to high‐grade dysplasia and invasive carcinoma after a period of time in which various somatic oncogenes can be mutated [3]. Management varies based on tumor size and involvement with the main pancreatic duct. Some patients with smaller, branch duct tumors are serially observed while larger, main duct tumors are intervened on surgically. PMP is a rare and serious presentation in which gelatinous fluid collections are associated with mucinous implants on the peritoneal surfaces and the omentum [4]. To date, PMP has been described in only 19 other cases, in the setting of IPMN. This phenomenon demonstrates a strong male predominance (15 males and 4 females). About 12 cases originated with cystic lesions in the tail of the pancreas (66%), five originated from the head (27%), one presented with diffuse disease, and the location was not reported in one case. Eight of these cases presented as a recurrence after a primary resection of an IPMN 6 months to 8 years after the initial resection. Nine cases presented with PMP at the time of their initial operation. Of those cases, six presented with diffuse mucinous ascites, two patients had extrapancreatic mucin confined to the lesser sac, and one presented with gastric and duodenal fistulae to a cystic pancreatic mass. Two cases presented with an IPMN that ruptured after initial finding prior to any surgical intervention. In the small number of cases reported, there is significant variation in presentation.

Our case is unique among these in that the patient did have a deposit of mucin in the pelvis but did not have diffuse peritoneal disease with significant mucinous ascites as described in the other reported cases. The three other cases that presented with PMP without diffuse disease all appear to be the result of direct extension, either into the lesser sac or invasion of the primary tumor into the stomach or duodenum. We did note some evidence of tumor rupture on the pathologic evaluation of our specimen with mucin in the lesser sac, however, the pelvic mucin deposit appeared separate with no direct communication from the lesser sac noted upon entry into the abdomen. This suggests that our patient was treated in the very early stages of disease dissemination. Furthermore, our case is the only case with a documented side‐branch origin for the IPMN. A single case has demonstrated a mixed ductal pathology, however, our pathologist review concluded there was no main duct involvement in the resected specimen.

Various mechanisms have been suggested for the pathogenesis of PMP from IPMN. In some cases, it is suggested that spillage during surgical resection is the source for a later recurrence. In the case of patients who present with PMP prior to any surgical intervention, as in our case, rupture of the cyst and dissemination of mucin‐producing cells throughout the abdomen is the suspected cause. The pathology of our resected specimen supports this theory, as acellular mucin extended beyond the capsule of the lesion and was surrounded by reactive mesenchymal cells, suggesting a rupture and resulting inflammation (Figure 1). Treatment and prognosis vary widely in the reported cases. Survival ranges from 3 months to greater than 6 years. Adjuvant chemotherapy was offered in some form to 11 of the 19 reported cases. Follow‐up among the reported cases is not consistent enough to determine if there is an association between adjuvant therapy and disease‐free survival. Six of the patients found to have PMP at the initial operation were treated with adjuvant chemotherapy. Cytoreductive surgery and/or heated intraperitoneal chemotherapy was also performed on seven patients.

With the significant variations in treatment and outcomes (Table 2) it is difficult to surmise a best practice in management. Several patients were not surgical candidates secondary to their comorbid illnesses. Long‐term survival is difficult to assess in the literature as there is significant variation, ranging from 3 months to greater than 7 years. Most patients presented with synchronous diffuse PMP but treatment varied widely in terms of operation performed and adjuvant therapy offered.

**Table 2:** Summary of cases reported in the literature with associated treatment and survival.

Study, year	Age/ sex	Tumor location	PMP presentation	PMP location	Tumor type	Operation	Adjuvant treatment	Survival
Hackeng et al. [5], 2019	62/M	Diffuse	Recurrence (48 month)^a^	Diffuse	Unk	TP/S	None	Not reported
Imaoka et al. [6], 2006	64/M	Tail	Synchronous	Diffuse	MD	DP	None	38 months
Imaoka et al. [7], 2012	74/F	Tail	Recurrence (12 month)	Diffuse	MD	1. DP 2. Resection of + margin	Gemcitabine	42 months
Imaoka et al. [7], 2012	56/M	Head	Recurrence (96 month)	Diffuse	MD	1. PD 2. TP/CRS	None	>48 months
Kataoka et al. [8], 2020	75/M	Body and tail	Progression of disease	Diffuse	MD	OMT/CRS	None	3 months
Kataoka et al. [8], 2020	51/F	Tail	Recurrence (24 month)	Diffuse	Mixed	1. DP 2. HIPEC 3. CRS	Gemcitabine, nab‐paclitaxel	Not reported
Kurihara et al. [9], 2000	74/M	Head	Recurrence	Diffuse	MD	PD	None	72 months
Lee et al. [10], 2007	55/M	Body	Synchronous	Diffuse	MD	DP/CRS	Gemcitabine, cisplatin	>3 months
Lee et al. [10], 2007	68/M	Head	Synchronous	DF, GF	MD	TP/S/PG	5‐FU based chemoradiation	>3 months
Mizuta et al. [11], 2005	53/M	Tail	Synchronous	Diffuse	MD	CRS/HIPEC	Gemcitabine	>24 months
Nepka et al. [12], 2009	82/M	Not reported	Synchronous	Diffuse	MD	None	None	>12 months
Rosenberger et al. [13], 2012	75/M	Tail	Synchronous	LS	MD	C/DP/S	None	>48 months
Rosenberger et al. [13], 2012	75/M	Head	Synchronous	LS	MD	C/PD	Palliative chemotherapy	43 months
Sanchez et al. [14], 2014	63/F	Tail	Recurrence (15 month)	Diffuse	MD	1. TP 2. CRS/HIPEC	Gemcitabine, cisplatin, radiotherapy	>70 months
Sirisai et al. [15], 2019	69/M	Tail	Synchronous	Diffuse	MD	1. DP/CRS/HIPEC 2. CRS/HIPEC	Proton beam radiation (52 Gy)	93 months
Sirisai et al. [15], 2019	54/M	Tail	Recurrence (6 month)	Diffuse	MD	1. DP 2. RS 3. CRS/HIPEC	1. S‐1 2. Irinotecan and S‐1	>33 months
Sirisai et al.[15], 2019	69/M	Tail	Progression of disease	Diffuse	MD	DP/S/CRS	Chemotherapy, unknown regimen	>7 months
Sugiura et al. [16], 2015	72/F	Body	Synchronous	Diffuse	MD	DP/S/CRS	S‐1	>12 months
Zanelli et al. [17], 1998	49/M	Head	Recurrence (60 month)	Diffuse	Unk	1. Puestow 2. PD 3. TP/CRS	5‐FU and carboplatin	>17 months
Presented case	65/F	Tail	Synchronous	LS and pelvis	BD	1. DP/S/CRS	None	—

*Note: Source*: [5–17]. LS, disease confined to lesser sac.

Abbreviations: BD, branch duct; C, cholecystectomy; DF, duodenal fistula; DP, distal pancreatectomy; GF, gastric fistula; MD, main duct; OMT, omentectomy; PD, Pancreaticoduodenectomy; PG, Partial Gastrectomy; S, splecectomy.

^a^Recurrence listed in months after initial operation.

Only one patient in the literature closely resembles our patient with localized mucin and no presence of concomitant adenocarcinoma on the final resection specimen. This patient was not treated in the adjuvant setting and was alive without evidence of recurrence at the most recent follow up (>48 months). Because of the similarities with this case, we have elected to follow our patient with close surveillance using CT imaging of the abdomen and pelvis at 6‐month intervals and defer chemotherapy at this time.

## 4. Conclusion

PMP arising from an IPMN of the pancreas is a rare and potentially devastating complication of the disease. Whether from rupture at the time of initial resection or rupture from progression of disease prior to intervention, this can present as a recurrence of disease or at initial presentation. Management tends to be extrapolated from practices surrounding appendiceal and ovarian sources of PMP. Given the complexity of these cases, a multidisciplinary discussion for ongoing management is essential, and these cases are best managed at centers with significant experience in the management of PMP from other sources.

## Funding

The authors have nothing to report.

## Conflicts of Interest

The authors declare no conflicts of interest.

## Supporting Information

Additional supporting information can be found online in the Supporting Information section.

## Supporting information


**Supporting Information** This article was prepared in accordance with the CARE Guidelines for case reporting. This internationally developed checklist is an effort to increase the accuracy, transparency, and usefulness of case reports. The completed CARE Checklist is included as a supporting file to this manuscript.

## Data Availability

Data sharing is not applicable to this article, as no new data were created or analyzed in this study.
